# Between Visible Marks and Invisible Pain: A Qualitative Study of Multi-Layered Stigma Among Women with Systemic Lupus Erythematosus in China

**DOI:** 10.3390/bs16060848

**Published:** 2026-05-26

**Authors:** Ning Xu, Hongzhe Xiang

**Affiliations:** 1 Faculty of Humanities and Arts, Macau University of Science and Technology, Macao SAR, China; 2 School of Journalism and Communication, Tsinghua University, Beijing 100084, China

**Keywords:** systemic lupus erythematosus, stigma, women’s health, invisible symptoms, workplace stigma, motherhood, qualitative study, China

## Abstract

Systemic lupus erythematosus (SLE) disproportionately affects women and often disrupts work, intimate relationships, and reproductive life. Although stigma is increasingly recognized in chronic illness, less is known about how it is experienced among women with SLE in China. Using an interpretive phenomenological design informed by health stigma theory, this qualitative study examined how stigma was produced, experienced, and managed in the everyday lives of 20 Chinese women living with SLE. Data were generated through semi-structured online interviews, chosen because of participants’ geographic dispersion and health-related constraints, and analyzed using an iterative thematic approach. The findings show that stigma operated through interconnected forms: visible bodily stigma linked to treatment-related appearance changes, invisible-symptom stigma that demanded ongoing credibility work, institutional stigma in employment, and gendered stigma in intimacy and motherhood. Participants responded through concealment, selective disclosure, and narrative labor: the work of presenting themselves as credible, responsible, and morally worthy despite social devaluation. These findings suggest that SLE stigma is not only interpersonal but also institutional and gendered, highlighting the need for stigma-sensitive clinical communication, workplace support, and reproductive counseling for women living with chronic illness.

## 1. Introduction

Health-related stigma is not simply a matter of individual prejudice. It is a power-laden social process in which labeling, stereotyping, separation, status loss, and discrimination become linked to power (Link & Phelan, 2001). This understanding provides the broad organizing lens for the present study: stigma is examined as a power-laden process through which illness becomes tied to diminished credibility, social value, and opportunity. Stangl et al.’s Health Stigma and Discrimination Framework further help locate this process across interpersonal, institutional, and structural levels, including everyday interaction, employment procedures, intimate relationships, and family expectations (Stangl et al., 2019). Goffman’s distinction between the discredited and the discreditable is especially useful for understanding chronic illnesses whose signs move between visibility and concealability. For Goffman, the discredited person bears a stigma that is already known or immediately apparent, whereas the discreditable person must manage information about a stigma that is not yet visible or fully known to others (Goffman, 1963). For people living with chronic illness, stigma can be especially consequential when symptoms are fluctuating, ambiguous, or not consistently visible, because they must repeatedly explain their condition while managing the risk of being judged as weak, unreliable, or personally at fault. In such cases, stigma management involves both managing social tension when illness is visibly marked and managing information when illness remains hidden or doubted.

Systemic lupus erythematosus (SLE) is a particularly important condition through which to examine chronic illness stigma. SLE is a chronic, relapsing, multi-system autoimmune disease that disproportionately affects women, especially during the reproductive years (Rees et al., 2017). Unlike conditions that are either clearly visible or wholly hidden, lupus often moves between visibility and invisibility. It may become visible through facial rash, swelling, hair loss, weight gain, or corticosteroid-related bodily changes, while some of its most disabling manifestations, such as fatigue, pain, and cognitive difficulty, remain difficult for others to see or verify (Chen et al., 2022; Jolly et al., 2012). This combination makes lupus especially vulnerable to stigma: patients may be marked when their bodies look “abnormal,” yet doubted when their suffering is not outwardly legible.

For women with SLE, stigma is further intensified by gendered social expectations. Body image disturbance is common in lupus and is associated with poorer psychosocial well-being and sexuality-related difficulties (Chen et al., 2022; Seawell & Danoff-Burg, 2005). Perceived illness stigma has also been linked to depression in women with SLE (Sehlo & Bahlas, 2013). These findings make it important to distinguish between different stigma mechanisms rather than treating stigma as a single psychological burden. Following Earnshaw and Chaudoir (2009), stigma may be enacted through direct or indirect devaluation, anticipated through expectations of future rejection, or internalized when devaluing beliefs are turned inward and become part of self-understanding. These distinctions are especially relevant for women with SLE because lupus stigma is not only biomedical; it is also tied to social norms that define a “good woman” as physically reliable, attractive, emotionally controlled, and potentially capable of marriage, childbearing, and caregiving. In this sense, the stigma attached to lupus is likely to extend beyond symptoms themselves to broader judgments about femininity, productivity, reproductive responsibility, and moral worth.

Existing lupus research has begun to recognize that stigma is not incidental to the illness experience. A thematic synthesis of qualitative studies found that adults living with SLE frequently described feeling misunderstood, disbelieved, and socially isolated (Sutanto et al., 2013). However, much of the existing literature still approaches the social consequences of SLE through relatively separate domains. For example, studies of body image have focused primarily on appearance-related distress, sexuality, and psychosocial well-being among women with SLE (Jolly et al., 2012; Seawell & Danoff-Burg, 2005), while workplace-oriented studies have examined employment barriers, work disability, disclosure, and the pressure to remain productive despite fluctuating symptoms (Bam & Lulema, 2025; Nowrouzi-Kia et al., 2025). Similarly, research on reproductive health has concentrated on fertility, pregnancy decision-making, disease management, and social support (Phuti et al., 2019; Alnaimat et al., 2025). These studies are valuable, but they often leave appearance-related concerns, invisible symptoms, employment difficulties, and reproductive issues analytically separated, rather than asking how they converge as a stigma process in everyday life. This is a crucial gap, because lupus stigma appears to operate simultaneously through bodily visibility, symptom illegibility, social devaluation, and gendered moral judgment.

The Chinese context makes this question especially important. Large-scale epidemiological evidence shows that SLE imposes a substantial and growing burden in China. A recent nationwide population-based study estimated that the standardized prevalence of SLE in urban China reached 47.61 per 100,000 overall and 94.16 per 100,000 among women, with age-specific incidence peaking at 30–49 years in women (Li et al., 2024). A national annual report further showed that the average age at diagnosis was 31.6 years and that nearly 80% of Chinese patients were diagnosed between 18 and 40 years old (Tian et al., 2022). In other words, lupus in China often unfolds during precisely those years when employment building, intimate partnership, fertility planning, and motherhood become socially salient. In a context where women’s social value remains strongly tied to bodily reliability, family responsibility, and reproductive expectations, stigma is likely to become a particularly consequential part of the illness experience.

However, Chinese research on SLE has only begun to address stigma directly. Recent studies have shown that perceived illness stigma exists among Chinese patients with SLE and is associated with psychological status (L. Zhang et al., 2024). Other Chinese work has examined social support, distress disclosure, and quality of life among women with SLE, demonstrating the importance of psychosocial and relational factors in their illness experience (Gao et al., 2022). Still, much of this literature treats psychological burden, social support, and stigma as separate outcomes. It tells us that women with SLE are distressed, but less often shows how stigma is actually produced through interaction, institutional rules, and gendered social expectations. In particular, there remains limited qualitative work in China examining how visible bodily changes, invisible symptoms, employment pressures, intimate relationships, and motherhood norms come together in women’s everyday lives.

Against this background, the present study examines how stigma is experienced, produced, and managed among women living with SLE in China. Drawing on qualitative interview data, it addresses three questions: (1) How do visible bodily changes and invisible symptoms become stigmatizing in everyday life? (2) How is stigma reproduced across institutional and relational settings, especially employment, intimacy, and motherhood? (3) How do women with SLE narrate, negotiate, and manage these stigmatizing experiences? By answering these questions, the article conceptualizes lupus stigma as a multi-layered and gendered social process rather than as a set of separate problems attached to appearance, symptoms, employment, or reproduction. In this usage, “multi-layered” refers to the interaction between bodily visibility, symptom invisibility, institutional exclusion, and gendered moral judgment across women’s everyday lives. It also develops the concept of narrative labor to describe the ongoing work through which women with SLE explain symptoms, manage disclosure, defend moral worth, and make stigmatized illness socially intelligible. This study therefore provides evidence that may inform more responsive clinical communication, psychosocial support, and stigma-sensitive interventions for women living with chronic illness.

## 2. Materials and Methods

### 2.1. Study Design

This article is based on data generated in a broader qualitative study of illness narratives among people living with SLE in China. The broader study was part of the first author’s doctoral research project and examined how patients interpreted illness, negotiated medical authority, and managed everyday life under conditions of chronic uncertainty. The present article is a focused secondary analysis of stigma-related material, with particular attention to women’s accounts of bodily change, symptom invisibility, employment, intimacy, and motherhood.

The present study used an interpretive phenomenological design. Interpretive phenomenology, rooted in hermeneutic phenomenology, examines how people make sense of lived experience within the social and relational worlds they inhabit (Heidegger, 1927/2010; van Manen, 2016; Laverty, 2003). This design was appropriate because stigma in SLE is not only an attitudinal phenomenon but also a lived and interpreted social process. It unfolds through bodily change, symptom doubt, concealment, disclosure, institutional exclusion, and gendered moral judgment. The design therefore allowed us to examine how women with SLE made meaning of being marked, doubted, judged, excluded, and required to defend moral worth in everyday life.

### 2.2. Participants and Inclusion Criteria

The broader study initially included 26 people living with SLE. After screening, 20 participants were retained for the present analysis. Four participants were excluded because they did not wish their interview materials to be included in research analysis, and two were excluded because they were men. The inclusion criteria were as follows: (1) a confirmed diagnosis of SLE for more than six months; (2) female; (3) aged 18 years or above; and (4) able to provide coherent oral accounts of illness-related experiences (see Table 1). The present article focuses on women because SLE disproportionately affects women and because stigma in this condition is deeply entangled with gendered expectations regarding appearance, work, intimacy, fertility, and caregiving.

Participants were recruited through patient networks, online communities, and snowball referrals. Sampling aimed to capture variation in age, marital status, employment status, disease duration, and life stage, while prioritizing information-rich cases relevant to the study’s interpretive aims rather than statistical representativeness. The final sample was judged sufficient on the basis of thematic saturation and information power. Evidence of saturation was observed during analysis: by the later interviews, core themes such as bodily self-discipline, symptom invalidation, employment exclusion, reproductive anxiety, and stigma management were recurring; additional interviews mainly added contextual variation rather than new analytic categories. Given the focused aim, specificity of the participant group, and depth of interviews, the sample was considered adequate for the present qualitative analysis (Malterud et al., 2016; Hennink & Kaiser, 2022).

### 2.3. Data Collection

The semi-structured in-depth interviews formed the core of the dataset because they enabled participants to describe illness trajectories, everyday challenges, and social interactions in their own terms. The interview guide covered diagnosis and treatment, bodily changes, symptom fluctuation, work and employment, family relationships, intimate partnerships, fertility and motherhood, and experiences of misunderstanding, exclusion, concealment, or disclosure.

The study received ethical approval from the Research Ethics Committee of the Faculty of Humanities and Arts at Macau University of Science and Technology (Certificate Number: MUST-FA-2025018). It was conducted entirely online in response to participants’ health conditions and geographical dispersion. Interviews were conducted in Mandarin Chinese. With participants’ consent, interviews were audio-recorded and transcribed verbatim. Nineteen interviews were fully audio-recorded; in one interview, recording was stopped at the participant’s request during discussion of sensitive experiences, and contemporaneous notes were taken with her permission. Analysis was conducted primarily on the Chinese transcripts. Quotations selected for publication were translated into English with the assistance of iFlytek translation software (v1.0), after key medical terms, colloquial expressions, and context-specific phrases had been checked by the authors. The corresponding author then reviewed the translated quotations against the original Chinese transcripts to preserve meaning, tone, and analytic relevance.

### 2.4. Data Analysis

The interpretive phenomenological design guided the analytic focus on participants’ meaning-making, while an iterative thematic approach provided the practical procedure for organizing and interpreting the data (Braun & Clarke, 2006; van Manen, 2016). First, all transcripts and field notes were read repeatedly to identify stigma-related passages, including accounts of being marked, doubted, judged, excluded, or forced to justify one’s illness. Second, these passages were coded comparatively, with attention to recurring patterns and cross-case variation related to life stage, illness duration, marital status, employment position, and social setting. Third, related codes were grouped into broader themes and reviewed against the dataset to ensure analytic consistency. Coding was conducted by the first author, who was also the interviewer and was therefore closely familiar with the broader narrative context of each account. Because the study used a reflexive thematic approach rather than a coding-reliability design, coding was not divided among multiple independent coders. Emerging codes, theme boundaries, and interpretive uncertainties were discussed with the corresponding author during the analytic process. Analytic memos were used to document coding decisions and to support reflexive comparison across cases (Braun & Clarke, 2019).

The analysis focused on how participants interpreted stigma across different but connected domains. Particular attention was paid to the interaction between visible bodily changes and invisible symptoms, and to how stigma extended beyond the clinic into employment, intimacy, and motherhood. The coding process also attended to participants’ own responses to stigma, including concealment, selective disclosure, bodily self-discipline, moral self-defense, and the use of peer support or narrative reframing. This allowed stigma to be analyzed not only as a form of social devaluation but also as a process that generated ongoing narrative and practical labor.

A shortened example of the coding structure used in the present article is shown in Table 2. A fuller version is provided in Appendix A (Table A1).

### 2.5. Reflexivity and Ethics

Because the study involved a population living with a long-term and potentially vulnerable condition, and because interviews addressed sensitive issues such as employment discrimination, relationship strain, reproductive anxiety, and social devaluation, ethical care and researcher reflexivity were integral to the research process. Participation was voluntary, and all participants were informed of the study purpose, their right to refuse any question, and their right to withdraw at any time without consequence. Pseudonyms are used throughout this article, and potentially identifying details have been altered or omitted.

The researcher approached interview accounts as narratives produced in interaction rather than as transparent reports. The interviews were conducted by the first author, a female researcher who was not involved in the participants’ medical care. This position may have made it easier for some participants to discuss appearance, intimacy, fertility, and relationship concerns outside a clinical relationship. At the same time, the researcher’s academic role and the interview setting may have shaped which experiences participants regarded as tellable or relevant. The corresponding author, a male researcher, was not the interviewer but participated in interpretive discussions during analysis. Because sexuality, intimacy, and reproduction were sensitive topics, the interviewer used open-ended prompts, allowed participants to decline or redirect questions, and avoided treating disclosure of such experiences as obligatory. In analyzing stigma, particular care was taken not to reduce participants to passive victims of prejudice, but to attend to how they themselves interpreted, negotiated, and managed stigmatizing experiences in everyday life.

## 3. Results

The results are organized around the meanings participants gave to stigma in different areas of everyday life. Across the themes, stigma was not experienced uniformly. While the movement between visible bodily marking and invisible-symptom doubt appeared across the sample, the salience of particular stigma experiences varied by life stage, illness duration, marital status, and employment position. Rather than treating these as fixed group differences, the analysis uses them to show how SLE stigma was shaped by participants’ changing social locations as well as by disease experience.

### 3.1. Visible Bodily Stigma and Altered Appearance

For many participants, stigma first became tangible through visible bodily change. In SLE, the body could suddenly become socially legible as “abnormal” through facial rash, swelling, weight gain, hair loss, acne, scarring, or corticosteroid-related bodily alterations. These visible changes were not experienced merely as unwanted clinical side effects. Rather, they became social signs that invited scrutiny, commentary, and judgment, especially in contexts where femininity remained closely tied to attractiveness, slimness, and self-control (Chen et al., 2022; Seawell & Danoff-Burg, 2005). In this sense, altered appearance became stigmatizing not simply because it made illness visible, but because it disrupted gendered expectations of bodily discipline, presentability, and feminine self-management (Bartky, 1990). In Goffman’s terms, these moments placed participants closer to the position of the discredited: the stigma was no longer fully concealable, because bodily change made illness or treatment visible before the participant could control how it was interpreted (Goffman, 1963).

Several women described corticosteroid-related bodily change as a turning point in how they were seen by others and by themselves. This turning point was most often located around the early period after diagnosis or during disease relapse, when higher doses of corticosteroids were used, and bodily changes such as weight gain, facial swelling, hair loss, acne, or skin changes became visible within a relatively short period. Some spoke of sudden changes in appearance as shocking not only because they altered the body, but because they made illness publicly visible. Treatment, in this sense, did not simply manage disease activity; it also transformed the body into a stigmatized surface.

*“I didn’t even dare go out. I felt people would think, ‘You’re already this fat, and you’re still eating?’”*

(P03)

P03’s account shows how anticipated stigma could become internalized as self-surveillance. She did not describe a specific insult in this moment; rather, she imagined how others might read her changed body and adjusted her behavior in advance. The imagined gaze of others was sufficient to produce withdrawal, shame, and spatial isolation. For participant P03, visible bodily change did not simply mean “looking different”; it meant becoming morally exposed. The expected judgment of being excessive or uncontrolled was turned inward, shaping how she saw and disciplined herself.

Other participants described the social consequences of becoming visibly “different” in school or family settings. Those who experienced dramatic treatment-related changes recalled being stared at, mocked, or treated as socially abnormal. Such experiences suggest that visible stigma in SLE was not reducible to the disease itself. Rather, it emerged through the interaction between illness, treatment, and dominant aesthetic standards. Prior work on lupus has similarly shown that body image disturbance is strongly associated with psychosocial distress among people living with SLE (Seawell & Danoff-Burg, 2005). In our data, however, appearance-related distress was not only personal or psychological. It was distinctly social: women were reacting to the ways their altered bodies were seen, interpreted, and ranked by others.

Participants responded through concealment and impression management. They wore masks, avoided photographs, declined social invitations, or explained visible changes as “allergies” or “skin problems” rather than naming lupus directly. Visible stigma therefore became an ongoing practical burden. It demanded continuous work to hide, soften, reinterpret, or distance the self from the bodily signs of illness. In this sense, the interactional task was not only to conceal information, but also to manage the tension produced when the body had already become socially marked.

### 3.2. Invisible Symptoms and Credibility Work

If visible bodily change marked women as abnormal, invisible symptoms forced them to prove that they were ill at all. Fatigue, pain, photosensitivity, and cognitive difficulties were among the most commonly described burdens, yet they were also the least easily recognized by others. Here, participants moved closer to Goffman’s position of the discreditable: because symptoms were not immediately visible, the central interactional problem became whether, when, and how to disclose illness, and how to make suffering credible once disclosed (Goffman, 1963). This pattern reflects a broader problem in chronic illness stigma: when suffering is not readily visible, patients are often required to justify its reality in interaction (Armentor, 2017).

Many women described a recurring contradiction. When lupus became visible, they were stigmatized as abnormal; when their symptoms were invisible, they were doubted as exaggerating, lazy, or emotionally fragile. Family members, classmates, or colleagues sometimes assumed that because women “looked fine,” they should still be able to work, socialize, or fulfill ordinary responsibilities without difficulty.

*“People would say, ‘You look fine, so why can’t you do it?’ But they couldn’t see how tired I was, how painful it was, or how hard it was just to get through the day.”*

(P06)

This kind of situation required what may be understood as credibility work: repeated efforts to explain why one could not do what others expected, even in the absence of visible signs. As a response to a discreditable condition, credibility work involved both information management and moral self-presentation. It can also be understood as part of the broader illness work (Strauss et al., 1985) required to manage chronic illness in everyday life, and as a form of identity work (Charmaz, 1983; Snow & Anderson, 1987) through which participants sought to sustain a credible and morally recognizable self despite being doubted. The burden was therefore not only informational, but moral. Participants felt that invisible suffering was frequently interpreted through a language of weakness or excuse-making rather than illness. In this sense, the invisibility of symptoms did not protect women from stigma; it often intensified suspicion.

Several participants described this explanatory labor as exhausting in its own right. To experience pain or fatigue was one burden; to repeatedly defend the legitimacy of that pain was another. This finding resonates with prior qualitative work showing that adults with SLE often feel misunderstood and disbelieved, especially because many central symptoms are socially difficult to verify (Sutanto et al., 2013). In our data, invisible symptoms therefore functioned as a second form of stigma, one that demanded constant translation of embodied suffering into socially credible language.

To make their illness believable, some participants tied their experiences to laboratory values, physicians’ orders, or treatment schedules. Rather than saying “I feel terrible,” they often felt compelled to say, in effect, “my indicators are unstable,” or “the doctor told me not to do this.” The authority of data and medical instruction became a way of defending the self against doubt. Invisible suffering thus pushed women into the role of witness, advocate, and translator of their own bodies.

### 3.3. Institutional Stigma as Procedural Exclusion in Employment

Stigma was also reproduced through institutional settings, especially employment. For many participants, the workplace was where illness became a practical and moral liability. In this context, institutional stigma took a procedural form: health examinations, hiring rules, recruitment screening, and assumptions about long-term reliability translated diagnosis into employment risk (Stangl et al., 2019). Exclusion did not require explicit hostility; it could be built into routine procedures that classified women with SLE as costly, unstable, or unreliable workers. These procedures were not gender-neutral. Feminist scholarship on gendered organizations and the “ideal worker” norm shows that workplaces often privilege bodies imagined as continuously available, predictably productive, and unencumbered by reproductive or caregiving responsibilities (Acker, 1990; Williams, 2000). This aligns with recent work showing that women with lupus often face a gendered continuum of concealment, reduced opportunity, and social devaluation in the workplace (Bam & Lulema, 2025).

Several participants identified employment physical examinations as a major threshold of exclusion. Lucy, for example, had originally planned to pursue postgraduate study in order to apply for civil service positions, but after her diagnosis, she realized that this pathway had effectively been closed to her.

*“I pursued graduate study because I wanted to take the civil service exam … but to be honest, lupus has a big impact on employment. Civil service recruitment completely bars people with lupus from applying. But from my medical report, my organs and body were actually fine.”*

(P15)

Participant P15’s account captures a distinction central to institutional stigma in employment: exclusion did not depend on actual work capacity, but on how the diagnostic label was processed within recruitment standards. She was not rejected because of a demonstrated inability, but because the condition had already been converted into an unacceptable employment risk. The physical examination therefore did more than assess health status; it functioned as a procedural sorting mechanism through which institutional standards turned diagnosis into a basis for preemptive exclusion, illustrating how stigma can become linked to status loss and discrimination through organizational power (Link & Phelan, 2001; Stangl et al., 2019). In this sense, stigma operated not through insult alone, but through formal standards and preemptive exclusion.

Others described subtler forms of workplace stigma. Employers rarely stated directly that lupus was the problem. Instead, exclusion was experienced as silence, evasiveness, or unexplained rejection.

*“I really had a hard time finding a job. I couldn’t choose the job; the job chose me. Employers would not tell you directly that you were unsuitable because of lupus. They would use other excuses, like saying the position had already been filled.”*

(P17)

P17’s account shows how institutional stigma could also be enacted through indirect and deniable recruitment practices. The problem was not only that employers might hold negative views of lupus, but that rejection could be routed through ordinary hiring language—“the position has already been filled”—which made exclusion difficult to identify, contest, or prove, a pattern consistent with research on subtle workplace discrimination and modern organizational discrimination (Jones et al., 2017; Hebl et al., 2020). This ambiguity was itself consequential. Even after entering work, participants continued to conceal diagnoses, minimize medical records, or carefully manage follow-up appointments so as not to appear unstable or costly. Some also lowered their career aspirations, seeking jobs with lower health demands and greater flexibility. In these narratives, lupus stigma was not simply a matter of colleagues’ attitudes; it was embedded in organizational norms regarding what a proper worker should look like, endure, and sustain. Employment stigma therefore operated as an institutional judgment about future risk, enacted through procedures and organizational ambiguity rather than only through interpersonal judgment about present illness.

### 3.4. Gendered Stigma in Intimacy and Motherhood

Among women with SLE, stigma became especially intense in intimate relationships and reproductive life. In these domains, gender did not simply intensify illness stigma; it shaped what became stigmatizing in the first place. Altered appearance, reproductive uncertainty, perceived infertility, and doubts about caregiving capacity became stigma markers because they were evaluated through expectations of femininity, marriageability, motherhood, and family responsibility. Participants therefore described being judged, or anticipating judgment, in ways that called into question their desirability, reproductive capacity, and reliability as future wives or mothers. These concerns echo research showing that reproductive uncertainty is a major psychosocial burden for women with SLE (Gao et al., 2022) and feminist scholarship on women’s responsibility for managing reproductive risk and family well-being (Waggoner, 2013; Waggoner, 2015).

For unmarried participants, they often framed lupus as a problem of disclosure, desirability, and future marriage: whether, when, and how to tell a (future) partner. Disclosure carried the risk of rejection; non-disclosure carried the risk of future blame. Yet this dilemma was intensified by the perception that lupus reduced one’s value within the marriage market. They feared being recast from “potential partner” to “problematic body.”

However, married or divorced participants more often described fertility, marital conflict, and family responsibility as the sites where stigma became consequential. Some participants recalled being told they might not be able to have children; others were not explicitly forbidden to become pregnant, but nevertheless came to experience themselves as potentially unfit mothers.

*“If I really got married and had a child, and that child also developed lupus, then I would feel I had wronged them. I shouldn’t have brought them into this world.”*

(P12)

This statement reveals how anticipated stigma could take the form of reproductive guilt. P12 was not responding to an existing accusation or an actual child’s illness; instead, she imagined a future in which her decision to have a child might be judged as irresponsible or harmful. Even before any actual harm occurs, she is positioned as someone who may fail at responsible motherhood. Such fears were not isolated individual thoughts; they were shaped by broader cultural expectations that women should protect future children from risk and embody reproductive responsibility.

For other participants, reproductive stigma was not only anticipated but became consequential within intimate relationships. P18’s account marks this shift from feared future judgment to experienced relational devaluation: perceived infertility became a basis for questioning marital legitimacy.

*“It was definitely because of the child issue—because I couldn’t have one.”*

(P18)

Her account shows how stigma can move from anticipated judgment to lived devaluation. In these narratives, lupus did not simply threaten reproductive plans; it transformed women into conditional partners whose legitimacy within marriage depended on demonstrating biological and moral womanhood. The stigma of illness thus merged with the stigma of perceived reproductive inadequacy.

Taken together, these accounts suggest that stigma in SLE is profoundly gendered. Women were not merely managing a chronic illness; they were confronting social scripts in which a proper woman is expected to remain physically presentable, emotionally stable, reproductively capable, and available for care work. Lupus disrupted these expectations simultaneously, which meant that illness-related changes were often interpreted as failures of femininity, partnership, motherhood, or care. As a result, stigma was experienced not only through external judgment but also through anticipated rejection, internalized shame, self-questioning, and anticipatory blame.

### 3.5. Narrative Labor: Managing Credibility, Disclosure, and Moral Worth

Participants were not passive recipients of stigma. Across settings, they developed practical and narrative strategies to manage how illness was seen, interpreted, and socially valued. We use the term narrative labor to describe this work: the ongoing effort to explain symptoms, calibrate disclosure, justify bodily limits, and present oneself as credible, responsible, and morally worthy despite social devaluation. This labor was “narrative” not because it consisted only of storytelling, but because participants repeatedly had to make illness intelligible to others and to themselves. It included concealment, selective disclosure, bodily self-discipline, moral self-defense, and the reframing of illness through peer recognition.

For participants closer to diagnosis or relapse, concealment was one of the most common responses. They often hid visible bodily changes where possible, avoided naming lupus directly, and carefully controlled who knew about the diagnosis. Yet concealment was rarely total. More often, they practiced selective disclosure, deciding who needed to know, how much to say, and what version of the illness to present. In contrast, participants with longer illness duration were not simply more “reconciled” to illness. Rather, they more often described accumulated repertoires of bodily and moral repair. Some tried to regain control over steroid-related bodily change through exercise, diet, or intensive self-discipline. Others emphasized being hardworking, considerate, and resilient in order to counter assumptions that they were burdensome or unreliable. Their illness narratives did more than describe symptoms; they actively defended a morally respectable self.

*“Even if I’m sick, I still want people to know that I’m trying my best to live properly, work properly, and not become a burden.”*

(P13)

This kind of self-presentation was central to stigma management. Women often responded not by openly confronting stigma, but by trying to preserve a version of themselves that could still be recognized as a good patient, a good worker, or a good woman. In this way, narrative labor became a key form of resistance as well as accommodation.

Peer support, especially online, also provided an alternative arena for stigma management. In patient communities, women could describe experiences without first having to prove that they were legitimate. These spaces did not erase stigma, but they enabled participants to move from defensive explanation toward mutual recognition. Prior studies of online health communities have similarly shown that sharing illness experiences can reduce isolation and help patients reinterpret suffering in more collective and less shame-laden terms (Ziebland & Wyke, 2012).

Taken together, these findings suggest that stigma in SLE is not only something women experience. It is also something they must continuously work on. Narrative labor captures this ongoing effort to protect the self from devaluation and to defend moral personhood in settings where illness was readily transformed into judgment.

## 4. Discussion

### 4.1. Main Contributions of the Study

By using an interpretive phenomenological design, this study shows how women with SLE made sense of stigma as neither singular nor incidental, but as a layered social process produced at the intersection of bodily appearance, symptom invisibility, institutional exclusion, and gendered moral expectations. Participants did not merely report that lupus was misunderstood; they described how it was rendered socially suspect in different ways across different settings. When bodily changes became visible, women were marked as abnormal, unattractive, or uncontrolled. When symptoms remained invisible, they were doubted, minimized, or treated as personally unreliable. In the workplace, lupus became a marker of future risk and diminished employability. In intimate and reproductive life, it was translated into questions of desirability, maternal fitness, and moral responsibility. These findings also show why it is useful to distinguish stigma mechanisms rather than treating stigma as a single psychological burden. Enacted, anticipated, and internalized stigma appeared in different forms across the data, but they often reinforced one another: experiences or expectations of devaluation shaped concealment, self-surveillance, reproductive guilt, and efforts to defend moral worth. Taken together, these findings suggest that stigma in SLE is not best understood as a single negative attitude toward disease, but as a socially organized process through which women’s bodies and biographies are reclassified as problematic.

A first contribution of this study is to show that lupus stigma operates simultaneously through visibility and invisibility. Existing scholarship on chronic illness stigma often treats visible and invisible conditions separately: visible difference may expose people to immediate labeling, while invisible conditions generate doubt and demands for proof (Armentor, 2017; Fitzgerald & Paterson, 1995). Building on Goffman’s distinction between the discredited and the discreditable, our findings suggest that SLE does not fit neatly into either side of this distinction (Goffman, 1963). Women with SLE may move between these positions across the course of illness and treatment: visible bodily changes can make them immediately discredited, while invisible fatigue, pain, or cognitive difficulty place them in the position of having to decide whether and how to disclose, explain, or justify illness. This dynamic echoes recent lupus research showing that patients’ lives are shaped by the temporality and context of fluctuating symptoms, including shame, masking, and efforts to minimize visible difference (Aim et al., 2022). The theoretical implication is that visibility and invisibility should not be treated only as fixed attributes of different illnesses or patient groups. In SLE, they operate as shifting and mutually reinforcing social positions: visible bodily change exposes women to judgment, while invisible symptoms require credibility work, and both shape the management of disclosure, appearance, and moral self-presentation.

A second contribution is to demonstrate the institutional dimension of SLE stigma. While much stigma research emphasizes interpersonal attitudes and social distance (Link & Phelan, 2001; Stangl et al., 2019), our findings show how stigma can be embedded in organizational procedures. Recruitment standards, employment physical examinations, and screening practices classified women with SLE as potential risks before work capacity was demonstrated. These mechanisms were often ambiguous and deniable: exclusion could occur through ordinary administrative procedures rather than explicit prejudice, making it difficult to identify, contest, or prove (Jones et al., 2017; Hebl et al., 2020). By connecting SLE employment experiences with research on subtle workplace discrimination, this study shows how institutional arrangements translate health status into anticipatory organizational disadvantage. This moves the analysis beyond employment difficulty toward a clearer account of how chronic illness stigma is produced at the institutional level.

A third contribution is to show that SLE stigma is gendered at the level of the categories through which illness is socially evaluated. Gender did not simply intensify an otherwise neutral illness stigma; it shaped what counted as a stigma marker in the first place. Altered appearance became stigmatizing because it disrupted expectations of feminine presentability and bodily self-management. Employment exclusion was shaped by an ideal-worker norm that privileges continuous availability, predictable productivity, and freedom from reproductive or caregiving responsibilities (Acker, 1990; Williams, 2000). In intimate and reproductive life, fertility, motherhood, relational dependability, and caregiving capacity became measures of feminine worth rather than merely biomedical concerns. The Chinese context gave these expectations particular familial and institutional force: in a post-one-child-policy era marked by pronatalist policy shifts and persistent family-centered expectations of marriage, childbirth, and intergenerational responsibility, reproductive uncertainty could be moralized as a failure of family duty rather than treated only as medical risk (Zeng & Hesketh, 2016; M. Zhang et al., 2024). In this sense, women were not only judged for being ill, but for appearing insufficiently attractive, reliable, employable, marriageable, fertile, or capable of care.

A fourth contribution lies in foregrounding narrative labor as a central response to layered stigma. Women with SLE did not merely suffer stigma; they worked on it through concealment, selective disclosure, symptom explanation, self-discipline, and moral self-defense. This concept builds on several established traditions without being reducible to any one of them. Like Goffman’s account of information control, narrative labor involves managing what others know and how they interpret a devalued condition (Goffman, 1963). Like illness work, it captures the everyday labor required to organize life around chronic illness (Strauss et al., 1985). Like identity work, it shows how people defend recognizable selves under devaluing conditions (Snow & Anderson, 1987; Charmaz, 1983). It also resonates with Hochschild’s emotional labor, as participants managed their feelings, expressions, and moral presentation to remain socially acceptable (Hochschild, 2012). Bury’s concept of biographical disruption is relevant as well, but narrative labor shifts attention from disruption itself to the continuing work of making a disrupted life morally intelligible (Bury, 1982). In SLE, narrative labor names the work of making a fluctuating, gendered, and often doubted illness intelligible as part of a credible and morally worthy life.

### 4.2. Implications for Intervention and Practice

These findings have several implications for intervention and practice. First, they highlight the need for more stigma-sensitive clinical communication. In SLE, distress is often intensified not only by disease activity but by misrecognition. Clinicians therefore need to recognize that non-visible symptoms such as fatigue, pain, and cognitive difficulty are not secondary complaints but central sites where legitimacy is negotiated. Clinical encounters that focus narrowly on laboratory indicators may unintentionally reinforce this problem by privileging what is measurable over what is lived. Interventions that integrate patient-reported symptoms, narrative check-ins, or structured opportunities to discuss work, body image, and reproductive concerns may help reduce the burden of self-justification and improve trust.

Second, the findings point to the need for workplace and policy interventions that move beyond generic anti-discrimination language. Participants’ accounts suggest that much stigma operates through ambiguity: selective screening, silent exclusion, fear of disclosure, and assumptions about reduced productivity. Practical interventions could include clearer employment protections for people with chronic episodic conditions, manager training on fluctuating illnesses, and workplace cultures in which accommodations do not automatically mark employees as weak or problematic. Recent work on invisible chronic illness at work similarly argues that safe disclosure and supportive managerial response are central to reducing both stigma and withdrawal (Nowrouzi-Kia et al., 2025).

Third, the results underscore the importance of gender-sensitive psychosocial and reproductive support. Women with SLE need not only medical advice about pregnancy, but also spaces in which fears of heredity, guilt, intimacy, and motherhood can be discussed without moral judgment. Counseling and care pathways that treat reproductive decision-making purely as risk management may fail to address the stigma and self-blame women actually carry. Interventions should therefore acknowledge that the reproductive burden of SLE is social and emotional as well as biomedical.

Finally, our findings support the value of peer and community-based support, including digital spaces, as sites of stigma repair. Online communities cannot eliminate stigma, but they may reduce isolation, provide alternative vocabularies for suffering, and lessen the need for women to constantly defend their legitimacy in unsympathetic environments. This is particularly relevant for conditions such as SLE, where daily life is shaped by uncertainty, variable visibility, and long-term negotiation between illness and ordinary social roles.

### 4.3. Limitations and Future Research

This study has several limitations. First, it is based on qualitative data from women living with SLE in China and does not aim for statistical generalizability. Its strength lies in analytic depth rather than prevalence estimation. Although sampling sought internal heterogeneity across age, marital status, employment status, educational background, illness duration, and region of residence, the sample may underrepresent women with lower educational attainment, women living in rural or digitally disconnected settings, women with severe disease who were unable to participate in lengthy interviews, and women who were not connected to patient networks or online communities. This recruitment strategy may also have shaped the stigma-management repertoires documented in the study, since women connected to patient communities may have had more opportunities to narrate illness, encounter peer vocabularies, or develop strategies for disclosure and self-defense than more isolated women. Second, because the present article is a focused analysis drawn from a broader qualitative project, the data were not originally collected solely for stigma research, although stigma-related themes were strongly present throughout the dataset. This design is well suited to examining how stigma emerged within broader illness narratives, but it cannot estimate the prevalence or relative weight of specific stigma experiences. Third, by centering women’s narratives, this article does not fully address how stigma may operate differently for men with SLE, family members, clinicians, or employers. Future research could build on these findings by comparing stigma management across gender, class, region, and occupational settings, and by examining how stigma is negotiated in clinical, workplace, and reproductive counseling interventions.

## 5. Conclusions

This study contributes to the literature by showing that stigma in SLE is multi-layered, institutional, and gendered. For women living with lupus in China, stigma is not simply a matter of being seen negatively because of illness. It is a process through which altered appearance, invisible symptoms, employment expectations, and motherhood norms become linked to moral evaluation. The concept of narrative labor captures the work women undertake to explain symptoms, manage disclosure, justify bodily limits, and preserve credibility and moral personhood under these conditions. Recognizing this complexity is essential if stigma-reduction efforts are to move beyond awareness campaigns and toward more responsive forms of clinical communication, workplace inclusion, reproductive counseling, and psychosocial support.

## Figures and Tables

**Table 1:** Participant Characteristics (N = 20).

Participant ID	Age Group	Illness Duration	Education	Occupational Category	Marital Status	Region of Residence
P01	35–39	11 years	Bachelor’s degree	Wellness	Single	Western China
P02	25–29	0.5 years	Master’s degree	Office/Technology	Single	Eastern China
P03	20–24	12 years	Bachelor’s degree	Student	Single	Western China
P04	25–29	1.5 years	Associate degree	Office/Technology	Single	Southern China
P05	20–24	1.5 years	Associate degree	Unemployed	Single	Southern China
P06	25–29	7 years	Bachelor’s degree	Office/Technology	Divorced	Central China
P07	20–24	1 year	Bachelor’s degree	Unemployed	Single	Northern China
P08	25–29	8 years	Bachelor’s degree	Education	Single	Central China
P09	25–29	10 years	Bachelor’s degree	Service	Single	Western China
P10	35–39	0.5 years	Associate degree	Self-employed	Married	Northern China
P11	25–29	9 years	Associate degree	Unemployed	Single	Western China
P12	25–29	9 years	Bachelor’s degree	Media/Communications	Single	Central China
P13	20–24	2 years	Master’s degree	Student	Single	Central China
P14	60–64	14 years	Not reported	Retired	Married	Southern China
P15	25–29	5 years	Associate degree	Unemployed	Married	Southern China
P16	40–44	14 years	Bachelor’s degree	Unemployed	Divorced	Northern China
P17	30–34	16 years	Bachelor’s degree	General employment	Single	Eastern China
P18	25–29	1 year	Doctoral degree	Student	Single	Eastern China
P19	25–29	1 year	Bachelor’s degree	Student	Single	Northern China
P20	35–39	21 years	Bachelor’s degree	Self-employed/Media	Married	Eastern China

**Table 2:** Representative Examples of Coding Framework for the Thematic Analysis.

Initial Codes	Focused Codes	Analytic Categories	Final Themes	Analytic Interpretation
Steroid-related weight gain, moon face, acne, hair loss, rash	Altered appearance as a stigma marker	Visible bodily stigma	Visible bodily stigma and altered appearance	Bodily changes were experienced not merely as side effects but as visible markers of abnormality that invited social judgment.
“You look fine,” fatigue not believed, pain doubted, brain fog difficult to explain	Symptom invalidation and credibility work	Invisible-symptom stigma	Invisible-symptom stigma and credibility work	Invisible symptoms required participants to make suffering socially credible and defend themselves against suspicion.
Failed employment physical examinations, hidden screening, reduced job opportunities	Diagnostic labeling and procedural exclusion	Institutional stigma	Institutional stigma as procedural exclusion in employment	Employment procedures could transform diagnosis into a marker of risk and preemptively restrict opportunity.
Fear of disclosure to partners, fear of heredity, feeling “unqualified” to become a mother	Moralization of reproductive risk	Gendered stigma	Gendered stigma in intimacy and motherhood	Reproductive uncertainty became stigmatizing because motherhood, fertility, and caregiving were treated as measures of feminine worth.
Selective disclosure, explaining symptoms, emphasizing effort, not wanting to be a burden	Moral self-defense and information control	Stigma management	Stigma management as narrative labor	Participants worked to present themselves as credible, responsible, and morally worthy despite social devaluation.

## Data Availability

The data presented in this study are available on request from the corresponding author. The data are not publicly available due to privacy or ethical restriction.

## Appendix A

**Table A1:** Example of Coding Framework for the Thematic Analysis (full version).

Initial Codes	Focused Codes	Analytic Categories	Final Themes	Analytic Interpretation
Steroid-related weight gain, moon face, acne, hair loss, rash, bodily “deformation”	Altered appearance as a stigma marker	Visible bodily stigma	Visible bodily stigma and altered appearance	Bodily changes are experienced not merely as treatment side effects but as visible markers of abnormality that invite social judgment, particularly by disrupting norms of femininity, bodily propriety, and social presentability.
Being stared at, talked about, mocked with nicknames, avoiding photographs, not daring to go out	Public scrutiny and appearance-based shame	Visible bodily stigma	Visible bodily stigma and altered appearance	Visibility exposes participants to public evaluation and comparison, which in turn produces social withdrawal, self-consciousness, and anticipatory shame.
“You look fine,” fatigue not believed, pain doubted, brain fog difficult to explain	Symptom invalidation and credibility crisis	Invisible-symptom stigma	Invisible-symptom stigma and credibility work	Invisible symptoms force participants to repeatedly prove that they are “really ill,” turning stigma into an ongoing burden of explanation and secondary injury.
Repeatedly explaining why one cannot stay in the sun, work overtime, or complete housework	Credibility work	Invisible-symptom stigma	Invisible-symptom stigma and credibility work	Participants rely on laboratory data, medical instructions, or bodily limits to secure recognition, but such repeated self-justification becomes a burden in itself.
Failed employment physical examinations, hidden screening by human resources, reduced job opportunities	Institutional consequences of diagnostic labeling	Institutional stigma	Institutional stigma in employment	Stigma is embedded not only in attitudes but also in formal procedures, recruitment criteria, and productivity norms that classify people with lupus as risky or unreliable workers.
Concealing medical history, paying out of pocket for examinations, avoiding medical insurance traces, remaining silent in job seeking	Strategic concealment under institutional risk	Institutional stigma	Institutional stigma in employment	Participants develop avoidance and concealment strategies in response to institutional rules in order to reduce the risk of exclusion, identification, or rejection.
Fear of disclosure to romantic partners, worry about being abandoned, being seen as unsuitable for marriage	Devaluation in the marriage and intimacy market	Intimacy-related stigma	Gendered stigma in intimacy and motherhood	Once lupus enters the sphere of intimacy, it is translated into judgments about desirability, burden, and long-term relational viability.
Fear of heredity, being told not to have children, feeling “unqualified” to become a mother	Moralization of reproductive risk	Reproductive/motherhood stigma	Gendered stigma in intimacy and motherhood	Women’s reproductive futures are moralized under conditions of chronic illness, and reproductive concerns are experienced as forms of stigma rather than merely medical uncertainty.
Feeling “sorry for the child,” “burdening the family,” “not being a good woman/good mother”	Internalized shame and anticipatory guilt	Reproductive/motherhood stigma	Gendered stigma in intimacy and motherhood	Stigma is internalized through gendered social norms, producing self-blame, shame, and anticipated failure even before explicit condemnation occurs.
Wearing masks, refusing photographs, selective disclosure, attributing symptoms to another illness	Information control	Stigma management strategies	Stigma management as narrative labor	Participants control visibility and information flow to reduce the risk of being marked, questioned, or devalued.
Emphasizing effort, discipline, and not wanting to be a burden	Moral self-justification	Stigma management strategies	Stigma management as narrative labor	Participants reconstruct themselves as responsible and disciplined subjects in order to counter stigma and maintain social legitimacy.
Seeking understanding in peer groups, finding similar others online, reinterpreting illness collectively	Peer recognition and meaning repair	Stigma management strategies	Stigma management as narrative labor	Peer communities provide alternative interpretive frameworks through which stigmatized experiences can be validated, normalized, and partially repaired.

Note. This table presents the analytic progression from initial stigma-related references in the data to focused codes, higher-order analytic categories, and final themes.
